# Supplementary material for: Predicting neutralization susceptibility to combination HIV-1 monoclonal broadly neutralizing antibody regimens
Source: bioRxiv. 2023 Dec 14:2023.12.14.571616. Preprint. [Version 1] doi: 10.1101/2023.12.14.571616 (PMC10760080; doi:10.1101/2023.12.14.571616)
Supplement: Supplement 1 [file NIHPP2023.12.14.571616v1-supplement-1.pdf]

Supporting information

Figure S1. Prediction performance for continuous (top row, CV R-squared) and binary (bottom row, CV AUC) neutralization outcomes for individual bnAbs (left-hand column) and the combination (right-hand column) BG1 + BG18 + NC37. For individual bnAbs, prediction performance is evaluated against the observed IC_{50} or IC_{80} values for the given bnAb; shapes denote the bnAb. For combination bnAbs, prediction performance is evaluated against both the observed IC_{50} or IC_{80} values based on the bnAb regimen (denoted by the prefix "observed") and the calculated combination IC_{50} or IC_{80} values based on the observed bnAb-specific values using the additive or Bliss-Hill method; shapes denote the combination method (additive or Bliss-Hill) and color denotes the approach (CP or PC). Error bars reflect 95% confidence intervals.

Figure S2. Prediction performance for continuous (top row, CV R-squared) and binary (bottom row, CV AUC) neutralization outcomes for individual bnAbs (left-hand column) and the combination (right-hand column) PG9 + PGT128. For individual bnAbs, prediction performance is evaluated against the observed IC_{50} or IC_{80} values for the given bnAb; shapes denote the bnAb. For combination bnAbs, prediction performance is evaluated against both

bioRxiv preprint doi: https://doi.org/10.1101/2023.12.14.571616; this version posted December 14, 2023. The copyright holder for this preprint (which was not certified by peer review) is the author/funder, who has granted bioRxiv a license to display the preprint in perpetuity. It is made available under aCC-BY 4.0 International license.

the observed IC_{50} or IC_{80} values based on the bnAb regimen (denoted by the prefix "observed") and the calculated combination IC_{50} or IC_{80} values based on the observed bnAb-specific values using the additive or Bliss-Hill method; shapes denote the combination method (additive or Bliss-Hill) and color denotes the approach (CP or PC). Error bars reflect 95% confidence intervals.

Figure S3. Prediction performance for continuous (top row, CV R-squared) and binary (bottom row, CV AUC) neutralization outcomes for individual bnAbs (left-hand column) and the combination (right-hand column) PG9 + PGT128 + VRC07. For individual bnAbs, prediction performance is evaluated against the observed IC_{50} or IC_{80} values for the given bnAb; shapes denote the bnAb. For combination bnAbs, prediction performance is evaluated against both the observed IC_{50} or IC_{80} values based on the bnAb regimen (denoted by the prefix "observed") and the calculated combination IC_{50} or IC_{80} values based on the observed bnAb-specific values using the additive or Bliss-Hill method; shapes denote the combination method (additive or Bliss-Hill) and color denotes the approach (CP or PC). Error bars reflect 95% confidence intervals.

Figure S4. Prediction performance for continuous (top row, CV R-squared) and binary (bottom row, CV AUC) neutralization outcomes for individual bnAbs (left-hand column) and the combination (right-hand column) PG9 + VRC07. For individual bnAbs, prediction performance is evaluated against the observed IC_{50} or IC_{80} values for the given bnAb; shapes denote the bnAb. For combination bnAbs, prediction performance is evaluated against both the observed IC_{50} or IC_{80} values based on the bnAb regimen (denoted by the prefix "observed") and the calculated combination IC_{50} or IC_{80} values based on the observed bnAb-specific values using the additive or Bliss-Hill method; shapes denote the combination method (additive or Bliss-Hill) and color denotes the approach (CP or PC). Error bars reflect 95% confidence intervals.

Figure S5. Prediction performance for continuous (top row, CV R-squared) and binary (bottom row, CV AUC) neutralization outcomes for individual bnAbs (left-hand column) and the combination (right-hand column) PGT128 + VRC07. For individual bnAbs, prediction

bioRxiv preprint doi: https://doi.org/10.1101/2023.12.14.571616; this version posted December 14, 2023. The copyright holder for this preprint (which was not certified by peer review) is the author/funder, who has granted bioRxiv a license to display the preprint in perpetuity. It is made available under aCC-BY 4.0 International license.

performance is evaluated against the observed IC_{50} or IC_{80} values for the given bnAb; shapes denote the bnAb. For combination bnAbs, prediction performance is evaluated against both the observed IC_{50} or IC_{80} values based on the bnAb regimen (denoted by the prefix "observed") and the calculated combination IC_{50} or IC_{80} values based on the observed bnAb-specific values using the additive or Bliss-Hill method; shapes denote the combination method (additive or Bliss-Hill) and color denotes the approach (CP or PC). Error bars reflect 95% confidence intervals.

Figure S6. Prediction performance for continuous (top row, CV R-squared) and binary (bottom row, CV AUC) neutralization outcomes for individual bnAbs (left-hand column) and the combination (right-hand column) VRC07-523-LS + PGDM1400. For individual bnAbs, prediction performance is evaluated against the observed IC_{50} or IC_{80} values for the given bnAb; shapes denote the bnAb. For combination bnAbs, prediction performance is evaluated against the calculated combination IC_{50} or IC_{80} values based on the observed bnAb-specific values using the additive or Bliss-Hill method; shapes denote the combination method (additive or Bliss-Hill) and color denotes the approach (CP or PC). Error bars reflect 95% confidence intervals.

Figure S7. Prediction performance for continuous (top row, CV R-squared) and binary (bottom row, CV AUC) neutralization outcomes for individual bnAbs (left-hand column) and the combination (right-hand column) VRC07-523-LS + PGT121. For individual bnAbs, prediction performance is evaluated against the observed IC_{50} or IC_{80} values for the given bnAb; shapes denote the bnAb. For combination bnAbs, prediction performance is evaluated against the calculated combination IC_{50} or IC_{80} values based on the observed bnAb-specific values using the additive or Bliss-Hill method; shapes denote the combination method (additive or Bliss-Hill) and color denotes the approach (CP or PC). Error bars reflect 95% confidence intervals.

Figure S8. Prediction performance for continuous (top row, CV R-squared) and binary (bottom row, CV AUC) neutralization outcomes for individual bnAbs (left-hand column) and the combination (right-hand column) VRC07-523-LS + PGT121 + PGDM1400. For individual bnAbs, prediction performance is evaluated against the observed IC_{50} or IC_{80} values for the given bnAb; shapes denote the bnAb. For combination bnAbs, prediction performance is evaluated against the calculated combination IC_{50} or IC_{80} values based on the observed bnAb-

bioRxiv preprint doi: https://doi.org/10.1101/2023.12.14.571616; this version posted December 14, 2023. The copyright holder for this preprint (which was not certified by peer review) is the author/funder, who has granted bioRxiv a license to display the preprint in perpetuity. It is made available under aCC-BY 4.0 International license.

specific values using the additive or Bliss-Hill method; shapes denote the combination method (additive or Bliss-Hill) and color denotes the approach (CP or PC). Error bars reflect 95% confidence intervals.

Figure S9. Prediction performance for continuous (top row, CV R-squared) and binary (bottom row, CV AUC) neutralization outcomes for individual bnAbs (left-hand column) and the combination (right-hand column) VRC07-523-LS + VRC26.25. For individual bnAbs, prediction performance is evaluated against the observed IC_{50} or IC_{80} values for the given bnAb; shapes denote the bnAb. For combination bnAbs, prediction performance is evaluated against the calculated combination IC_{50} or IC_{80} values based on the observed bnAb-specific values using the additive or Bliss-Hill method; shapes denote the combination method (additive or Bliss-Hill) and color denotes the approach (CP or PC). Error bars reflect 95% confidence intervals.

Figure S10. Prediction performance for continuous (top row, CV R-squared) and binary (bottom row, CV AUC) neutralization outcomes for individual bnAbs (left-hand column) and the combination (right-hand column) 3BNC117 + PG9. For individual bnAbs, prediction performance is evaluated against the observed IC_{50} or IC_{80} values for the given bnAb; shapes denote the bnAb. For combination bnAbs, prediction performance is evaluated against both the observed IC_{50} or IC_{80} values based on the bnAb regimen (denoted by the prefix "observed") and the calculated combination IC_{50} or IC_{80} values based on the observed bnAb-specific values using the additive or Bliss-Hill method; shapes denote the combination method (additive or Bliss-Hill) and color denotes the approach (CP or PC). Error bars reflect 95% confidence intervals.

Figure S11. Prediction performance for continuous (top row, CV R-squared) and binary (bottom row, CV AUC) neutralization outcomes for individual bnAbs (left-hand column) and the combination (right-hand column) 10-1074 + 3BNC117. For individual bnAbs, prediction performance is evaluated against the observed IC_{50} or IC_{80} values for the given bnAb; shapes denote the bnAb. For combination bnAbs, prediction performance is evaluated against both the observed IC_{50} or IC_{80} values based on the bnAb regimen (denoted by the prefix "observed") and the calculated combination IC_{50} or IC_{80} values based on the observed bnAb-specific values using the additive or Bliss-Hill method; shapes denote the combination method (additive or Bliss-Hill)

bioRxiv preprint doi: https://doi.org/10.1101/2023.12.14.571616; this version posted December 14, 2023. The copyright holder for this preprint (which was not certified by peer review) is the author/funder, who has granted bioRxiv a license to display the preprint in perpetuity. It is made available under aCC-BY 4.0 International license.

and color denotes the approach (CP or PC). Error bars reflect 95% confidence intervals.

Figure S12. Prediction performance for continuous (top row, CV R-squared) and binary (bottom row, CV AUC) neutralization outcomes for individual bnAbs (left-hand column) and the combination (right-hand column) 10-1074 + 3BNC117 + PG9. For individual bnAbs, prediction performance is evaluated against the observed IC_{50} or IC_{80} values for the given bnAb; shapes denote the bnAb. For combination bnAbs, prediction performance is evaluated against both the observed IC_{50} or IC_{80} values based on the bnAb regimen (denoted by the prefix "observed") and the calculated combination IC_{50} or IC_{80} values based on the observed bnAb-specific values using the additive or Bliss-Hill method; shapes denote the combination method (additive or Bliss-Hill) and color denotes the approach (CP or PC). Error bars reflect 95% confidence intervals.

Figure S13. Prediction performance for continuous (top row, CV R-squared) and binary (bottom row, CV AUC) neutralization outcomes for individual bnAbs (left-hand column) and the combination (right-hand column) 10-1074 + 10E8 + 3BNC117. For individual bnAbs, prediction performance is evaluated against the observed IC_{50} or IC_{80} values for the given bnAb; shapes denote the bnAb. For combination bnAbs, prediction performance is evaluated against both the observed IC_{50} or IC_{80} values based on the bnAb regimen (denoted by the prefix "observed") and the calculated combination IC_{50} or IC_{80} values based on the observed bnAb-specific values using the additive or Bliss-Hill method; shapes denote the combination method (additive or Bliss-Hill) and color denotes the approach (CP or PC). Error bars reflect 95% confidence intervals.

Figure S14. Prediction performance for continuous (top row, CV R-squared) and binary (bottom row, CV AUC) neutralization outcomes for individual bnAbs (left-hand column) and the combination (right-hand column) 10-1074 + PG9. For individual bnAbs, prediction performance is evaluated against the observed IC_{50} or IC_{80} values for the given bnAb; shapes denote the bnAb. For combination bnAbs, prediction performance is evaluated against both the observed IC_{50} or IC_{80} values based on the bnAb regimen (denoted by the prefix "observed") and the calculated combination IC_{50} or IC_{80} values based on the observed bnAb-specific val-

bioRxiv preprint doi: https://doi.org/10.1101/2023.12.14.571616; this version posted December 14, 2023. The copyright holder for this preprint (which was not certified by peer review) is the author/funder, who has granted bioRxiv a license to display the preprint in perpetuity. It is made available under aCC-BY 4.0 International license.

ues using the additive or Bliss-Hill method; shapes denote the combination method (additive or Bliss-Hill) and color denotes the approach (CP or PC). Error bars reflect 95% confidence intervals.

Figure S15. Prediction performance for continuous (top row, CV R-squared) and binary (bottom row, CV AUC) neutralization outcomes for individual bnAbs (left-hand column) and the combination (right-hand column) 10E8 + 3BNC117. For individual bnAbs, prediction performance is evaluated against the observed IC_{50} or IC_{80} values for the given bnAb; shapes denote the bnAb. For combination bnAbs, prediction performance is evaluated against both the observed IC_{50} or IC_{80} values based on the bnAb regimen (denoted by the prefix "observed") and the calculated combination IC_{50} or IC_{80} values based on the observed bnAb-specific values using the additive or Bliss-Hill method; shapes denote the combination method (additive or Bliss-Hill) and color denotes the approach (CP or PC). Error bars reflect 95% confidence intervals.

Figure S16. Prediction performance for continuous (top row, CV R-squared) and binary (bottom row, CV AUC) neutralization outcomes for individual bnAbs (left-hand column) and the combination (right-hand column) 10E8 + 3BNC117 + PG9. For individual bnAbs, prediction performance is evaluated against the observed IC_{50} or IC_{80} values for the given bnAb; shapes denote the bnAb. For combination bnAbs, prediction performance is evaluated against both the observed IC_{50} or IC_{80} values based on the bnAb regimen (denoted by the prefix "observed") and the calculated combination IC_{50} or IC_{80} values based on the observed bnAb-specific values using the additive or Bliss-Hill method; shapes denote the combination method (additive or Bliss-Hill) and color denotes the approach (CP or PC). Error bars reflect 95% confidence intervals.

Figure S17. Prediction performance for continuous (top row, CV R-squared) and binary (bottom row, CV AUC) neutralization outcomes for individual bnAbs (left-hand column) and the combination (right-hand column) 10E8 + PG9 + PGT128. For individual bnAbs, prediction performance is evaluated against the observed IC_{50} or IC_{80} values for the given bnAb; shapes denote the bnAb. For combination bnAbs, prediction performance is evaluated against both the observed IC_{50} or IC_{80} values based on the bnAb regimen (denoted by the prefix "observed") and

bioRxiv preprint doi: https://doi.org/10.1101/2023.12.14.571616; this version posted December 14, 2023. The copyright holder for this preprint (which was not certified by peer review) is the author/funder, who has granted bioRxiv a license to display the preprint in perpetuity. It is made available under aCC-BY 4.0 International license.

the calculated combination IC_{50} or IC_{80} values based on the observed bnAb-specific values using the additive or Bliss-Hill method; shapes denote the combination method (additive or Bliss-Hill) and color denotes the approach (CP or PC). Error bars reflect 95% confidence intervals.

Figure S18. Prediction performance for continuous (top row, CV R-squared) and binary (bottom row, CV AUC) neutralization outcomes for individual bnAbs (left-hand column) and the combination (right-hand column) 10E8 + PGT128 + VRC07. For individual bnAbs, prediction performance is evaluated against the observed IC_{50} or IC_{80} values for the given bnAb; shapes denote the bnAb. For combination bnAbs, prediction performance is evaluated against the calculated combination IC_{50} or IC_{80} values based on the observed bnAb-specific values using the additive or Bliss-Hill method; shapes denote the combination method (additive or Bliss-Hill) and color denotes the approach (CP or PC). Error bars reflect 95% confidence intervals.
